# How to Achieve Fast Entrainment? The Timescale to Synchronization

**DOI:** 10.1371/journal.pone.0007057

**Published:** 2009-09-23

**Authors:** Adrián E. Granada, Hanspeter Herzel

**Affiliations:** Institute for Theoretical Biology, Humboldt-Universität zu Berlin, Berlin, Germany; University of Glasgow, United Kingdom

## Abstract

Entrainment, where oscillators synchronize to an external signal, is ubiquitous in nature. The transient time leading to entrainment plays a major role in many biological processes. Our goal is to unveil the specific dynamics that leads to fast entrainment. By studying a generic model, we characterize the transient time to entrainment and show how it is governed by two basic properties of an oscillator: the radial relaxation time and the phase velocity distribution around the limit cycle. Those two basic properties are inherent in every oscillator. This concept can be applied to many biological systems to predict the average transient time to entrainment or to infer properties of the underlying oscillator from the observed transients. We found that both a sinusoidal oscillator with fast radial relaxation and a spike-like oscillator with slow radial relaxation give rise to fast entrainment. As an example, we discuss the jet-lag experiments in the mammalian circadian pacemaker.

## Introduction

Biological rhythms are ubiquitous in nature and are found in diverse systems, from spiking neurons to animal populations with periods ranging from milliseconds to years. Our everyday life exhibits many behavioral and physiological oscillations that interact with the external fluctuating environment. Biological pacemakers typically interact with other oscillators including for example coupled rhythms of heart, respiration and movement [1], vocal fold oscillations [2] and singing duets of birds [3]. These interactions can lead to mutual synchronization as in the collective blinking of fireflies [4] and entrainment in which oscillators synchronize to a common signal. An example of this is the left and right birdsong control nuclei HVc that show synchronization in the absence of interhemispheric connections [5]. Another example is the entrainment of plant-leafs movements to the light-dark and cold-warm cycles [6]. Complex interactions between multiple oscillators are observed in the mammalian suprachiasmatic nucleus (SCN), where mutual synchronization and entrainment are combined. These tiny nuclei situated in the anterior hypothalamus are responsible for controlling endogenous circadian rhythms. Many different body functions like sleep-wake cycles and body temperature rhythms are regulated by centrally generated neuronal and hormonal activities. The SCN consists of two nuclei of about ten thousand densely packed neurons and generates a stable robust period of about 24 h. The SCN has the striking ability of fast reentrainment as observed in jet-lag type experiments, where after an abrupt phase shift of 6 h, the SCN can be almost completely reentrained within one cycle [7]–[9]. Also from the induced loss of rhythmicity in SCN slices after application of tetrodotoxin (TTX, a voltage gated sodium channel blocker), the SCN cells resynchronize within one cycle [10]. When TTX is applied, the oscillations are lost at a single cell level but after washing TTX out, the cells start oscillating again in a synchronized manner after 1 day. Such short transients times are remarkable, bearing in mind the large number of coupled oscillators involved and the diversity of their initial conditions and periods [11], [12]. How synchronization and entrainment mechanisms work within the SCN neurons is one of the main open problems in the field of circadian rhythms. Furthermore, in jet-lag and shift work schedules, the reentrainment time is of major relevance and has been associated to a number of diseases, ranging from sleep disorders to cancer [13]–[15].

Several mathematical models of SCN cells have been proposed with an increasing complexity (using 7 up to 73 differential equations [16]–[18]), none of which describes the short reentrainment times in detail. Our goal in this present paper is to unveil the specific dynamics that can lead to ultrafast entrainment. We present a generic model to characterize transient times leading to entrainment. This model is governed by two basic properties of the oscillator: (a) the radial relaxation timescale and (b) the phase velocity distribution around the limit cycle. When an oscillator is perturbed, the radial relaxation timescale determines the rate of convergence back to the unperturbed amplitude and it can hence be associated with robustness towards amplitude fluctuations. The phase velocity distribution determines the waveform of the oscillation.

Studying the transient time as a function of these two properties will give us a general understanding of how fast can entrainment be reached. Those two basic properties are inherent in every oscillator and, therefore, such a concept can be applied to many biological systems to predict the transient time to entrainment. Even more interestingly, one can infer properties of the underlying oscillator from the observed transient times as we will show later. Helpful insights derived from transients of an oscillatory system have already been applied in heart cells studies [19]. Although we focus on biological applications, the presented theory can be applied to many other oscillatory systems undergoing entrainment.

## Results

### Timescales of entrainment

**Figure 1:**
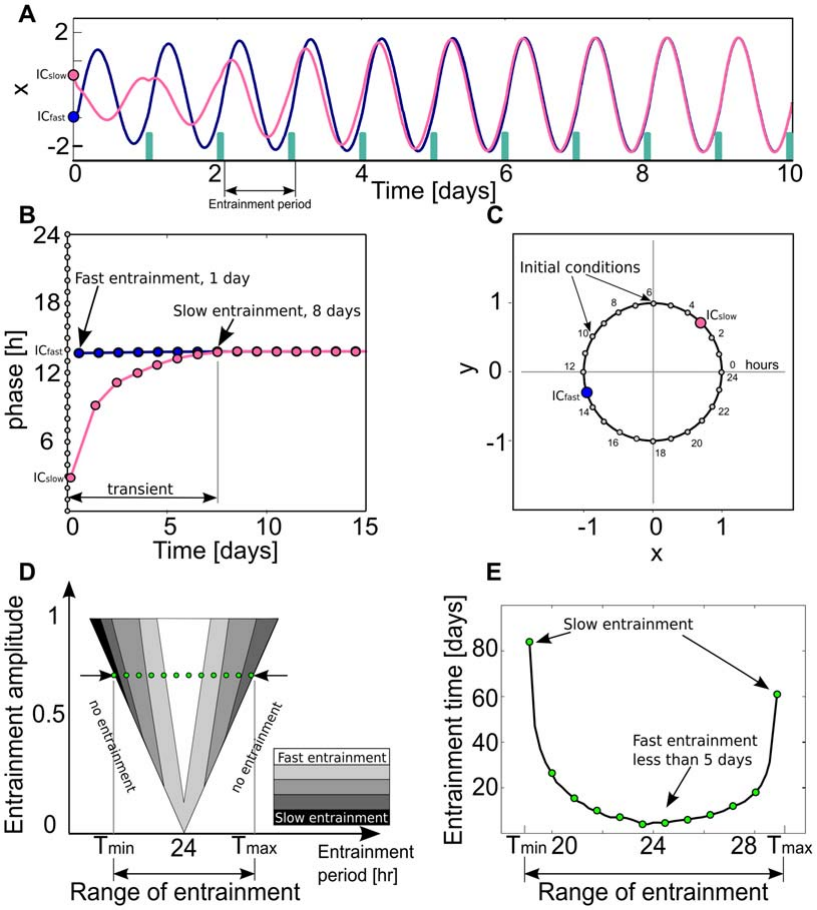
Basic mechanisms involved in the entrainment of an oscillator. (A) Time series for two initial conditions, ICslow and ICfast, leading to a long transient (pink) and a short transient (blue), respectively. The green bars represent the entrainment signal. (B) Phase evolution for both initial conditions. ICfast entrain after 1 day while IC slow needs 8 days (fig. 1) (C) Oscillator limit cycle representation with 24 marked initial conditions (gray). (D) Schematic representation of the entrainment region as a function of the entrainment amplitude and period (often termed 1∶1 “Arnold tongue”). Gray scale represents different transients to entrainment zones within the entrainment region. The green dots represent the section of the entrainment region (entrainment range) for a certain entrainment amplitude and in (E) their associated entrainment times are plotted as a function of the entrainment period. Computational details of A,B and E are given in Materials and Methods.

When a system is entrained, it reaches a stable phase relation with the external rhythm and thus their phase difference becomes constant (see Materials and Methods). The transient time it takes to reach this stable phase relation depends on the initial conditions (ICs), the entrainment signal and the properties of the oscillator. An example of these transients for a generic circadian oscillator is shown in Figure 1. Each initial condition has an associated initial phase (see gray dots in Figure 1B and C), different initial phases can lead to big differences in the transient time to entrainment. Figure 1A shows the time evolution of two initial conditions, 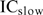 and 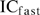, leading to a long transient (pink) and a short transient (blue) respectively. This can also be observed in a phase evolution plot where 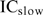 needs 8 days to achieve a stable phase relation whereas 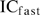 only 1 day (see Figure 1B). The dependence on initial conditions for a specific circadian oscillator model has already been studied [20]. A self-sustained oscillator is able to entrain just to certain combinations of entrainment amplitudes and periods that define the so-called entrainment region or 1∶1 “Arnold tongue” (see Figure 1D). In other words, each entrainment amplitude entrains the system within a certain period range, from a minimum 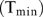 to a maximum 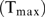 entrainment period, known as range of entrainment. Typically, at the borders of this entrainment region the transients leading to entrainment are much longer than those at the center (see Figure 1E). In the following we focus on those inherent properties of the oscillator that determine the transients.

### Generic oscillator model

As will be seen, our results indicate that two characteristics of the oscillator determine the transients: the radial relaxation time and the phase velocity around the limit cycle. To better illustrate the dependence of transient times on these two properties, we introduce a simple model oscillator that can mimic various oscillators. We use a generic circular oscillator of radius 1 and period 1 (arbitrary units) so the results can be easily rescaled to other systems. As a specific model, we introduce a variation of the Poincaré oscillator [21], given by
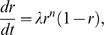
(1)

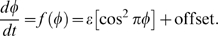
(2)


**Figure 2:**
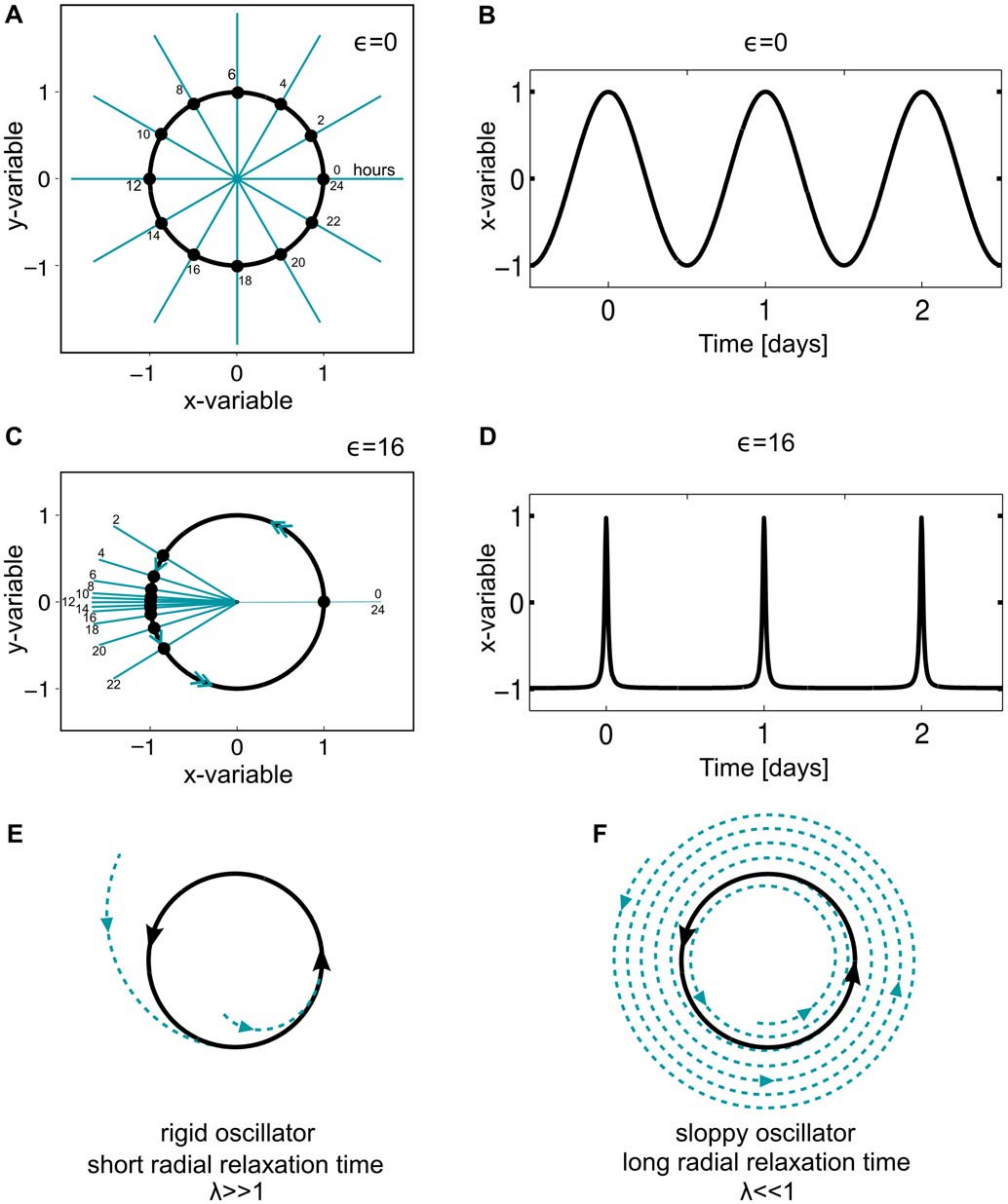
Limit cycle representations in phase space for a sinusoidal and spike-like oscillator together with their time series. (A) Sinusoidal oscillator: limit cycle with 12 marked phase points (dots) and isochrons (rays). The intersection of each isochron with the limit cycle determines the phase. (B) Temporal evolution of 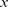 variable with parameters 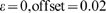. (C) Spike-like oscillator, where most isochrons are concentrated in a small region of the limit cycle. (D) Temporal evolution of the 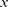 variable with parameters 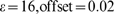. Representations of both oscillators (sinusoidal or spike-like) with short radial relaxation time (E) and long radial relaxation time (F) are also shown. Computational details are given in Materials and Methods.

This oscillator can be smoothly switched from a sinusoidal shape to a spike-like oscillator, while the radial relaxation can be independently controlled (see Figure 2). Equation 1 describes the radial evolution and has a stable orbit at 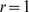, with a radial relaxation controlled by the parameters 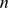 and 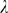. For 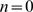 the radial relaxation is exponential and for 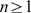 the radial relaxation is nonlinear. For 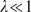 the radial relaxation time, 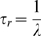, is long and for 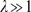 the radial relaxation time is short, sometimes referred to as “sloppy” and “rigid” oscillators respectively (see Figure 2E and F). Equation 2 describes the phase evolution or, in other words, the velocity around the limit cycle, where 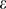 controls the velocity difference between the fastest (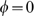) and slowest (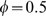) points. The “offset” is a small positive constant and guarantees that the velocity is never zero (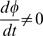). For 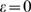 (i.e., 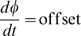), there are no velocity variations along the limit cycle, and the oscillator is sinusoidal (see Figure 2A and B). For 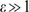, we have large velocity differences along the limit cycle, leading to a spike-like behavior (see Figure 2C and D).

We use this generic limit cycle model instead of the widely used phase models because amplitude dynamics will be of fundamental importance in characterizing transients leading to entrainment. The phase velocity around the limit cycle determines the temporal shape of the oscillation (waveform), as illustrated in Figure 2B and D. The radial relaxation rate 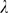 together with the degree of nonlinearity controlled by the parameter 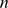 determines the timescale of convergence of perturbed solutions to the limit cycle (see Figure 2E and F). It can be associated with robustness towards amplitude fluctuations.

This oscillator, a modified Poincaré oscillator [21], belongs to the class of radial isochron limit cycles (RILC) due to its radial symmetry (see Materials and Methods). Many examples of useful biological insights based on RILC's can be found elsewhere [1], [21], [22]. Here we use the Winfree definition of isochrons as lines in phase space leading to the same asymptotic phase. Thus all initial conditions located on the same isochron will reach the limit cycle with the same phase [23]. The intersections of the isochrons and the limit cycle trajectory are the temporal phase points (see the dots in Figure 2A and C). In the case of RILCs, the isochron structure in the whole phase space can be deduced from the distribution of temporal phases. Thus a sinusoidal oscillator has equally distributed phase points and isochrons (see Figure 2A). A spike-like oscillator, on the other hand, makes a rapid excursion along the fast branch to spend most of its time at the slow branch. This time scale separation generates an asymmetric distribution of isochrons at the limit cycle by compressing them around the slow branch (see Figure 2C). The isochron distribution will be essential for the general understanding of the transient time to entrainment. As mentioned, our model was designed such that the phase velocity around the limit cycle and the radial relaxation time can be independently controlled to explore their influence on transients. For clarity, the oscillator will be rescaled to a period of 1 day and entrained with pulse-like perturbations of 1 h length. Square waveform oscillators, like the van der Pol oscillator in the relaxation regime, are not captured by this 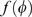. In order to simulate square waveform oscillators a new 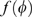 is introduced (see Figure S1 in Supporting Information).

### Median time to entrainment

The time to entrainment depends strongly on the period ratio of external and internal rhythms (detuning) and on the initial phase (ICs). The internal period, such as the free-running period in circadian biology, and phase of the oscillators are typically unknown or difficult to measure. Therefore, we minimize the effects of detuning and initial conditions by studying ensembles of different external periods and initial phases. This allow us to associate a characteristic 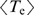 with specific properties of an oscillator. The median time to entrainment 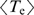 is the median value of 12 different 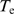 s. We use 12 different external periods evenly distributed within the range of entrainment to calculate the median as shown in Figure 1D and E. Additionally, for each external period, 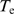 is taken as the median time from 24 uniformly distributed initial temporal phases (see Figure 1D). By taking both medians, we reduce the dependence on initial condition and entrainment period significantly (see Materials and Methods).

**Figure 3:**
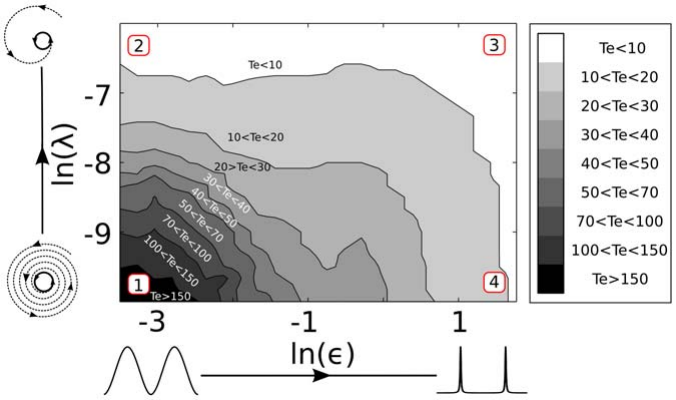
Median times to entrainment 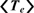 as a function of the phase velocity distribution around the limit cycle 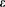 and the radial relaxation constant 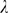. Gray scale encodes the time to entrainment where black represents long 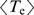 and white represents short 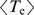. Both axes are plotted on logarithmic scales and 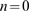 in Equation 1. Computational details are given in the Materials and Methods.

We start our results discussion with the exponential radial relaxation case (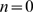 in Equation 1) and describe the nonlinear case at the end of this section. For the case of exponential relaxation, we calculate the time to entrainment 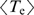 for different oscillator types using a broad range of values of phase velocity parameters 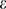 and of radial relaxation rates 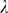, such that 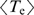 shows significant variations (see Figure 3). The entrainment signal was generated with short and medium-sized square periodic pulses. Specifically, we used 1 h pulse length with an amplitude of 0.8. This leads to a “range of entrainment” similar to that observed in rat locomotor activity under light pulse entrainment 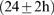 [24]. As mentioned above, the “range of entrainment” refers to the range of periods that a self-sustained oscillator is capable of entraining by a 1∶1 frequency ratio.

**Figure 4:**
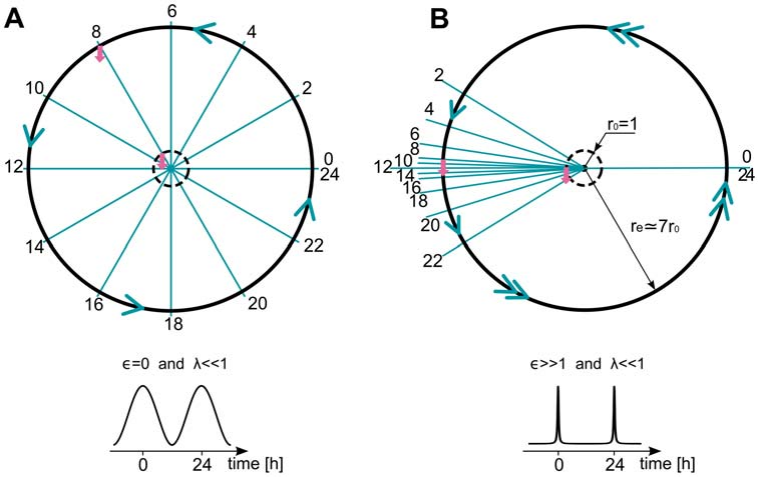
Representative sinusoidal and spike-like limit cycles with long radial relaxation time. Isochrons are represented as thin rays and perturbation pulses as pink arrows. (A) Unperturbed sinusoidal limit cycle trajectory (dashed small circle) and the expanded entrained limit cycle (solid large circle) for the sinusoidal oscillator. Initially the pulse generates a phase change up to 4 h, but later the pulse phase shift is reduced to less than 0.5 h. (B) Unperturbed spike-like limit cycle trajectory (dashed small circle) and the expanded entrained limit cycle (solid big circle) for the spike-like oscillator. Initially the pulse generates phase advances up to 14 h and, after some pulses, the phase shifts are still 8 h. The original limit cycle (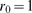) expands here about 7 times. The lower panels show the characteristic time series pattern of a sinusoidal and spike-like oscillator.

According to Figure 3, the longest time to entrainment is found when the limit cycle has a sinusoidal temporal pattern and if the radial relaxation time is long (box 1 in Figure 3). The radial relaxation time, 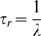, is in this case much longer than all other involved time scales: external periods 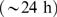, endogenous period (24 h) and pulse duration (1 h). Such long radial relaxation times allow the entrainment pulses to considerably perturb the trajectory of the limit cycle, leading to an expanded entrained orbit (a representative scheme of the mechanism is shown in Figure 4). Between the pulses, however, the system has not enough time to relax back to the original unperturbed orbit. A sinusoidal oscillator (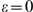) implies equally distributed isochrons along the limit cycle and thus all isochrons diverge symmetrically from the limit cycle center. While the limit cycle expands, the isochrons spread apart, so phase changes induced by the same pulse size decrease. The phase change induced by a single pulse can be deduced from the difference between the starting isochron, where the perturbation starts, and the final isochron, where the trajectory is located after a given perturbation (see pink arrows in Figure 4). The combination of limit cycle expansion and equally distributed isochrons reduces the effect of each pulse. Very few isochrons are crossed leading to smaller phase changes per pulse. Consequently, it a rather long time to reach the final stable phase. For illustrative purposes we use vertical pulses in Figure 4, but similar features are observed with other types of entrainment signals.

Surprisingly, the median time to entrainment 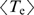 can be reduced up to 12-fold in our parameter range by changing independently 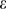 or 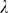. Keeping 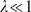 but increasing 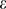 smoothly, changes the sinusoidal waveform oscillator into a spike waveform oscillator (see box 4 in Figure 3). The spike-like oscillator is also known as relaxation oscillator due to its fast and slow branches. The oscillator spends most of its time on the slow branch, so most stimuli are received on this branch. Spike-like oscillators have lower isochron divergence angles from the origin (see Figure 4B). This small isochron divergence allows considerable phase shifts of pulses despite the expansion of the limit cycle. This isochron clustering and, consequently, their low divergence angles allow the system to reach the final stable phase much faster.

As shown in Figure 3, an increase in the relaxation rate 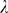 leads to a drastic reduction in the median transient time 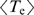 as well. In this case, the radial relaxation time is much shorter than the period keeping the trajectory to the unperturbed limit cycle with keeping the trajectory to the unperturbed limit cycle with 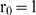. Thus pulses induce considerable phase shifts for every given pulse and phase shifts are not reduced due to limit cycle expansion (compare box 2 in Figure 3).

The spike-like oscillator with short radial relaxation time is optimal as far as time to entrainment is concerned, because isochrons are concentrated in the slow branch without suffering from limit cycle expansion (see box 3 in Figure 3). In this case, perturbations induce large phase jumps, leading quickly to a stable phase from almost any initial condition.

In the present example, we used entrainment pulse amplitudes of 0.8, but qualitatively similar results are also observed with smaller amplitudes and also with sinusoidal perturbations (see Figure S2 in Supporting Information). As shown in Figure S2, the entrainment signal amplitude and waveform do not play a major role in determining the transient to entrainment.

Oscillators with highly nonlinear radial relaxation exhibit a much shorter median time to entrainment 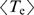 as shown in Figure S2C and D in Supporting Information. This property is captured by our model using 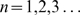 in Equation 1. The normal form of limit cycles arising via supercritical Hopf bifurcations corresponds to 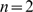. Due to this strong nonlinearity, perturbations to the limit cycle trajectory relax rapidly back to the unperturbed limit cycle reducing considerably the limit cycle expansion effect (see Figure S2D). In the following, we relate our theory to a specific biological rhythm to gain insight into the properties of the system.

### Fast entrainment in the mammalian circadian pacemaker

Physiological and behavioral processes in most organisms are synchronized with a 24 h day-night rhythm. Mammals have a central pacemaker located in the hypothalamic suprachiasmatic nucleus (SCN) that orchestrates circadian rhythms for the whole body. The SCN consists of two nuclei of about ten thousand densely packed neurons and generates a stable robust period of circa 24 h. This stable neuronal and hormonal rhythm regulates many different body functions. Cells within the SCN have an endogenous molecular clock based on a network of interlocking feedback loops of genes and proteins. The intercellular coupling between individual neurons generates not only a robust 24 h collective self-sustained rhythm under constant conditions (complete darkness) but also confers robustness against mutations [25]. The suprachiasmatic nucleus has a heterogeneous complex architecture. There is spatial heterogeneity, and individual neurons differ in their neuropeptide expression, light responsiveness, phase, and free running period [11], [12], [26]. For example, individual periods of dispersed cells span over 20 to 30.9 h with an average period of 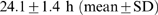. In organotypic slice cultures, periods range from 22.4 to 26.7 h with an average of 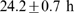 [12]. Surprisingly, despite this complexity, the SCN exhibits fast reentrainment. In jet-lag type experiments the SCN can be almost completely reentrained within one day after an abrupt phase shift of 6 h [7]–[9]. Advanced microscopic techniques allow single cell bioluminescence measurements of clock proteins at intervals as short as 20 min. These measurements display almost sinusoidal oscillations [10], [25]. However, bioluminescence measurements provide only smoothed time series of specific reporter constructs and thus it is not entirely clear how sinusoidal the underlying core oscillator is. From our generic model, we predict that the observed fast reentrainment can be achieved in the following ways: (i) Sinusoidal waveform oscillator with relative short radial relaxation times (box 2 in Figure 3); (ii) spike-like oscillations with long relaxation time (box 4 in Figure 3); or (iii) a spike-like waveform and short radial relaxation time (box 3 in Figure 3). If SCN cells are self-sustained sinusoidal oscillators, we predict that the SCN cell oscillators have a short radial relaxation time. The radial relaxation time can be experimentally determined via a nonlinear fit to a time series in which an amplitude relaxation can be observed as in Figure 1A. The nonlinear fit can be done with the ansatz 
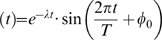
, where 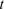 is time, 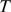 is the oscillation period, 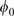 the initial phase difference and 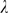 is the radial relaxation from which the radial relaxation time 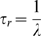 can be directly obtained. In the vicinity of the limit cycle, the radial relaxation rate can be directly connected to the Floquet exponents. Large Floquet exponents (short radial relaxation times) have already been predicted on the basis of robustness studies using different clock models [27] and by optimizing a specific feedback model [28]. Our generic approach is based on one single characteristic, namely, the transient time to entrainment, and thus our prediction is independent of specific model assumptions. Most SCN cell models assume self-sustained oscillation, but experimental data [29], [30] and theoretical predictions [31], [32] suggest an alternative scenario, where most SCN cells might behave as damped oscillators. Detailed characterization of the transient time to entrainment with a mixture of sustained and damped oscillators is beyond the scope of the present work.

### The Goodwin oscillator

The Goodwin oscillator [33] is a minimal model that describes the oscillatory negative feedback regulation of a protein which inhibits its own transcription. It provides a basic description of the central components in the circadian oscillators of Neurospora, Drosophila, and mammals [31], [34]. In this model, a clock gene mRNA (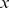) produces a clock protein (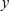) which, in turn, activates a transcriptional inhibitor (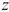). Here we study a version of the Goodwin oscillator successfully used to model data from dermal fibroblasts from skin biopsies of human subjects [35]. The aim of that study was to investigate whether different types of behavior, early (“larks”) or late chronotypes (“owls”), have different clock properties in dermal fibroblasts. The model equations are:
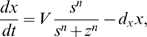
(3)

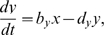
(4)

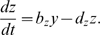
(5)


**Figure 5:**
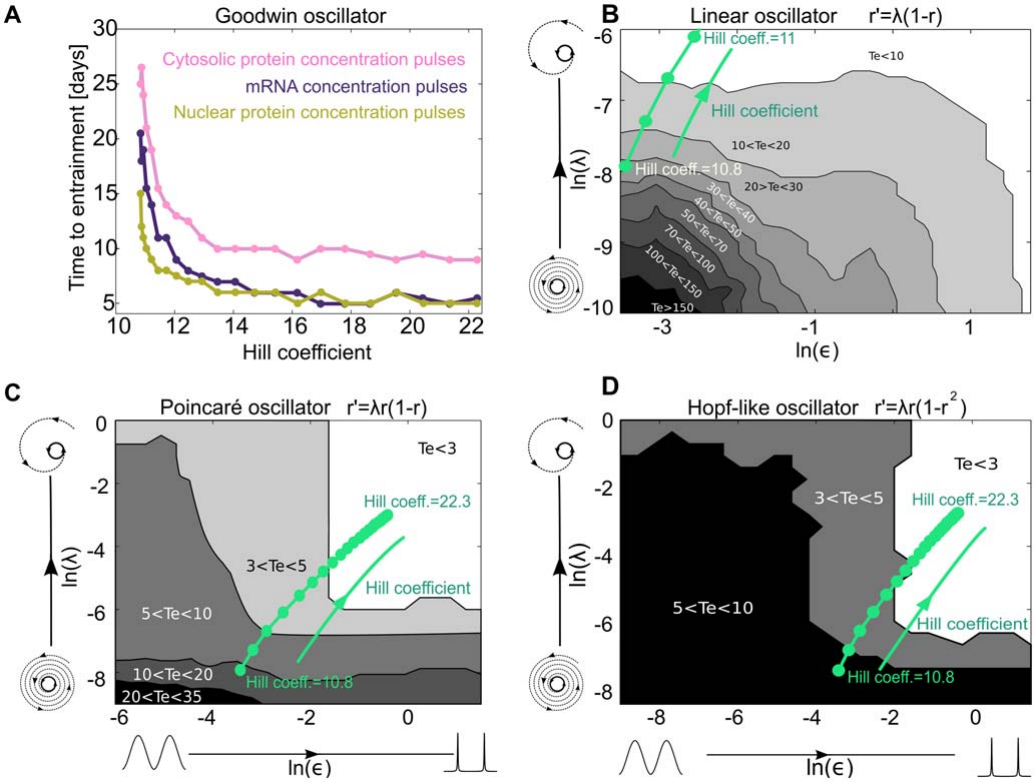
Median time to entrainment 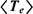 for the Goodwin model and the comparison with three radial oscillators models. (A) Median time to entrainment for the Goodwin oscillator as a function of the Hill coefficient. 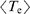 was calculated for an entrainment signal applied separately to each variable: the cytosolic protein concentration (pink), the mRNA concentration (dark violet) and the nuclear protein concentration (brown). (B) 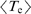 for a linear oscillator and the values of 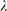 and 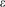 extracted from the Goodwin oscillator (green). (C) 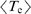 for a Poincaré oscillator and the values of 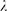 and 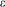 extracted from the Goodwin oscillator (green). (D) 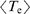 for a Hopf-like oscillator and the values of 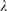 and 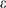 extracted from the Goodwin oscillator (green). 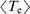 was calculated as in Figure 3 with relative pulse strength 0.4.s.

This model describes the time evolution of mRNA (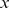), of a cytosolic clock protein (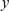) and a nuclear clock protein (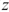). Concentrations of these are measured in arbitrary units (a.u.). This model has mostly linear kinetics with production rate constants 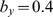 and 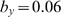 and degradation rate constants 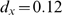, 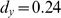, and 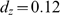, all rate constants in 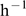. The Hill function, which expresses the transcription rate that is inhibited by the nuclear clock protein (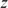), has a maximal rate 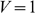 a.u./h, a half-saturation point 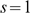 a.u., and a Hill coefficient which we vary between 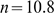 and 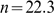. Our aim is to compare our generic predictions discussed above with a biochemical oscillator model. All eight parameters have an influence on the dynamics of the system. It has been shown that the Hill coefficient has a strong influence on the oscillator properties [35]. Therefore, we choose the Hill coefficient as the parameter to calculate the associated time to entrainment 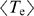. Indeed, simulations confirm that the Hill coefficient has a strong effect on the time to entrainment. In Figure 5A, an increase from 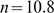 to 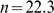 reduces the time to entrainment 5-fold. The entrainment signal was applied to all three dynamical variables in turn: to the cytosolic protein concentration, to mRNA concentration and to nuclear protein concentration. We observe qualitatively similar results in all three cases. Furthermore, in order to relate these transient times to our generic models, we extract for each Hill coefficient in the Goodwin model the velocity variations along the limit cycle parameter 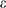 and the Floquet exponent associated with the radial relaxation timescale parameter 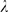. In this way, we can project these values on our plots for three models of radial oscillators (see Figure 5B, C and D). Interestingly, the Hill coefficient changes both velocity variations along the limit cycle 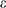 and the radial relaxation timescale 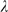. Importantly, these two parameters govern the transients also in this higher-dimensional biochemical model. This is a demonstration that biochemical models are amaneable for studies using the concept developed in this paper.

In addition to the results presented above we also checked if our findings hold for square-waveform oscillators, alternative entrainment signals and the more general scenario of mutually coupled oscillators (see Figures S1, S2 and S3 in Supporting Information). In all three cases we obtained qualitatively similar results in agreement with our concept.

## Discussion

To our knowledge, ours is the first study that characterizes the transient time to entrainment in terms of the oscillator properties. Entrainment can be regarded as a particular case of synchronization with unidirectional coupling between the oscillators. Therefore, similar features observed in our results might be expected in other synchronization scenarios (see Supporting Information S1). The time to synchronization for a network of oscillators has been studied for several systems using analytical approaches [36]–[40] and numerical simulations [41]–[43]. Most synchronization studies focused on specific model oscillators at the network level and derived scaling laws associated with the number of oscillators. In [41] and [43], the synchronization rate of different conductance based models (Hodgkin-Huxley type models) was studied. Both studies showed that, a spike-like oscillator reached a synchronized state much more rapidly than a sinusoidal oscillator. Interestingly, it has been shown that synchronization can be achieved in a few cycles by relaxation oscillators [44] and by more sinusoidal “repressilators” [45]. Generally in models, the radial relaxation time and the phase velocity cannot be controlled independently. Therefore, changing the waveform pattern generally also changes the radial relaxation time, compounding the contributions of both properties and confusing the interpretation. Indeed, we observed this in the case of the Goodwin oscillator while increasing the Hill coefficient (see Figure 4C). Our goal was to reach a general understanding of the transient to entrainment based on topological representations. We use numerical simulations to exemplify our basic ideas. The model independent results can be related to most previously conducted studies.

Under the assumption that SCN cells are self-sustained sinusoidal oscillators, we predict that single cell oscillators have a short radial relaxation time. However, we cannot exclude that some SCN cells are spike-like oscillators and exhibit short transients this way. In fact, each SCN cell is a complex molecular oscillator and certain variables might exhibit a sinusoidal shape while others might have a spike-like shape. Perhaps, the pathway governing transients might be associated with a spike-like components. Time scale separations that support this view can be inferred from the rapid reentrainment observed in the SCN. Experiments with light pulses show that some core components of the SCN are able to respond to light within 1 h [46], [47].

In summary, we have shown how the time to entrainment is governed by the interplay of the radial relaxation time and the phase velocity distribution around the limit cycle. The time to entrainment 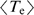 might be considered as an essential dynamical feature of an oscillator. In many systems, this quantity can be more easily extracted from experimental data than other related dynamical features such as Floquet exponents or isochron distributions. The median transient time to entrainment can be used to infer properties of the underlying oscillator from the observed transient times.

## Materials and Methods

### Model oscillator

**Figure 6:**
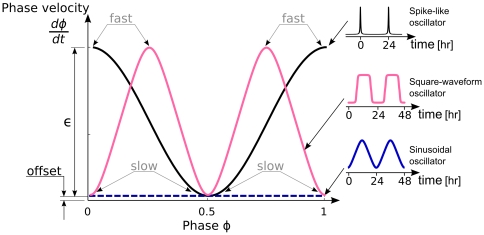
Phase velocity 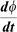 as a function of the phase for different 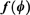. 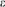
 controls the velocity gap between the fastest and slowest points and the parameter “offset” guarantees that the velocity is never zero. The black line corresponds to a spike-like oscillator, the pink line corresponds to the square-waveform oscillator and the dashed blue line corresponds to a sinusoidal oscillator with a constant phase velocity around the limit cycle.

The oscillator was designed to explore how the median time to entrainment 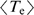 depends on a few generic parameters that are applicable to a big class of oscillators. In Equation 2, 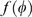 describes the phase evolution, where the parameter 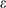 controls the ratio between the slowest and fastest velocities around the limit cycle. For 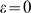, 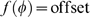 results in a sinusoidal oscillation (dashed blue line in Figure 6), for 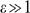, a spike-like oscillation is generated (black line in Figure 6) and for a new 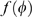 we obtain a square-waveform oscillator (pink curve in Figure 6). The parameter 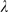 controls the radial relaxation time independently of the phase dynamics. In the vicinity of the limit cycle, 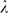 can be associated with the Floquet exponents, and 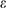 with the isochron structure [23] of the limit cycle. This model allows us to create a spike-like oscillator with arbitrary Floquet exponents.

A modified Poincaré oscillator is also known as radial isochron limit cycle due to the radial structure of its isochrons. The phase dynamics 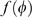 is independent of the radial variable 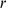. Isochrons can be analytically calculated in some simple cases [21] or otherwise extracted with numerical approaches [48], but these approaches are not needed here since the isochrons can be projected directly from the temporal phase points plotted in Figure 3. Isochrons are a powerful tool to understand the phase changes induced by perturbations [49].

The simulations were carried out using the equations 1 and 2 with 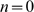 and in Cartesian coordinates:
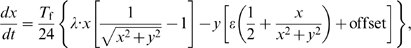
(6)

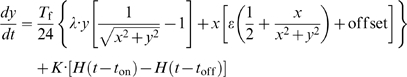
(7)


Here 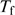, the unscaled period, depends on the parameters 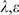 and offset. As discussed below we rescaled the period to 24 h by choosing an appropriate scaling factor 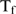. The entrainment signal 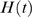 is the Heaviside step function, 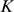 is the pulse strength, the pulse start time is 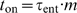, with 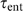 the entrainment period, 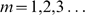 and 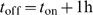 is the pulse end time.

### Time to entrainment

Our numerical experiments were designed to reduce dependencies on initial conditions and entrainment frequency. In Figure 3 we calculated the 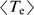 for a wide range of 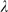 and 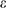 values. Each point of the plot was calculated following the same numerical protocol: 1) Choose a parameter combination (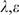) of interest and rescale the system to a period 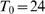 h. 2) Calculate the range of entrainment and choose entrainment frequencies equidistributed within this range. 3) Choose initial temporal phases 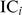. The 24 h temporal initial phases are located around the unperturbed limit cycle (gray dots in Figure 1B and C), i.e. each initial condition is given by 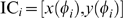, where 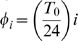 with 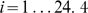. 4) Start the simulations with periodic 1 h vertical pulses and calculate the instantaneous phase difference between the oscillator and the train of pulses for a total duration of 500 days (see Figure 1B). 5) The time to entrainment is considered to be reached if the mean phase difference of eight consecutive cycles is smaller than 5 minutes. Otherwise, no entrainment is detected. 6) Repeat steps 3–5 for the 24 different temporal phase initial conditions and then take their median value Te (see Figure 1C). 7) Repeat steps 2–6 for 12 evenly distributed frequencies within the total range of entrainment and then take their median value 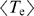 (see Figure 1E). 8) Choose another combination of 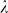 and 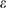 and restart the protocol.

## Supporting Information

Supporting Information S1 (0.06 MB PDF)

Figure S1 Square waveform oscillator, its time series and the median time to entrainment. (A) Square waveform oscillator: limit cycle with 24 marked phase points (dots) and isochrons (rays). The intersection of each isochron with the limit cycle determines the phase and (B) the temporal evolution of x variable with parameters ε = 1, offset = 0.02, n = 0 and (C) the median time to entrainment <T_e_> as a function of the phase velocity around the limit cycle, ε, and radial relaxation constant, λ, for pulse entrainment. Gray scales refer to the median time to entrainment, where black represents long and white short <T_e_>.
(0.72 MB TIF)

Figure S2 Median time to entrainment <T_e_> for different entrainment signals and oscillators, under soft-pulses entrainment and under medium-sized-pulses for a nonlinear oscillator and for a Hopf oscillator. (A) Entrainment under sinusoidal perturbations with amplitude 0.05. (B) Entrainment under pulse perturbation with amplitude 0.4. (C) Entrainment under 1 h pulse perturbation with amplitude 0.8 for a nonlinear radial relaxation oscillator. (D) Entrainment under 1 h pulse perturbation with amplitude 0.8 for a Hopf oscillator. The median time to entrainment is plotted as a function of the phase velocity around the limit cycle, ε, and radial relaxation constant, λ. Gray scales refer to the median time to entrainment, where black represents long and white short <T_e_>. Both axes are plotted using logarithmic scales.}
(1.23 MB TIF)

Figure S3 Time to synchronization for two coupled oscillators. (A) Time to synchronization of two coupled “sloppy’ oscillators as a function of their transition from sinusoidal to a spike-like oscillator (B) Time to synchronization of two sinusoidal oscillators as a function of their transition from “sloppy’ to “rigid’ oscillator. See Supporting Information for model details.}
(0.83 MB TIF)
