# Supplementary material for: How to Achieve Fast Entrainment? The Timescale to Synchronization
Source: PLoS One. 2009 Sep 23;4(9):e7057. doi: 10.1371/journal.pone.0007057 (PMC2745570; doi:10.1371/journal.pone.0007057)
Supplement: Supporting Information S1 — (0.06 MB PDF) [file pone.0007057.s001.pdf]

Supporting Information

Square waveform oscillators

Square waveform oscillators, like the van der Pol oscillator in the relaxation regime, cannot be described by $f(\phi)$ presented in Equation 2. Therefore, we introduce a new function $f(\phi)$ given by

$$\frac{dr}{dt} = \lambda r^n (1 - r), \tag{S1}$$

$$\frac{d\phi}{dt} = f(\phi) = \frac{\epsilon}{2}\left[1 - \cos\frac{\pi\phi}{6}\right] + \text{offset}. \tag{S2}$$

For clarity, the oscillator is rescaled to a period of 24 h. Equation S1 describes the radial evolution and has a stable orbit at $r = 1$, with a radial relaxation controlled by the parameters n and λ. Equation S2 describes the phase evolution for a 24 h oscillator where ϵ controls the velocity difference between the fastest, at $\phi = 6$ and $\phi = 18$, and slowest at $\phi = 0$ and $\phi = 12$ (see Figure S1). The "offset" is a small positive constant and guarantees that the velocity is never zero ($\frac{d\phi}{dt} \neq 0$). For $\epsilon = 1$ we have large velocity differences along the limit cycle, leading to a square waveform oscillator. The longest median time to entrainment, $\langle T_\mathrm{e} \rangle$, is found when the oscillator has a sinusoidal temporal pattern (see box 1 in Figure S1C). Keeping $\lambda \ll 1$ but increasing ϵ smoothly changes the sinusoidal waveform oscillator into a square waveform oscillator (see box 3 in Figure S1C). The square waveform oscillator is also known as relaxation oscillator due to its fast and slow branches. The oscillator spends most of its time on the slow branches, around $\phi = 0$ and $\phi = 12$ so most stimuli are received on these branches. Square waveform oscillators have, as the spike-like case, a small isochron divergence that allows considerable phase shifts of pulses despite the limit cycle expansion effect. So as in the previous cases, the isochron clustering and, consequently, their low divergence angles allow the system to reach the final stable phase much faster. An increase in the relaxation rate λ leads to a drastic reduction in the median transient time $\langle T_\mathrm{e} \rangle$ as well. In this case, the radial relaxation time is much shorter than the period keeping the trajectory to the unperturbed limit cycle with $\mathrm{r}_0 = 1$. Thus pulses induce considerable phase shifts for every given pulse and phase shifts are not reduced due to limit cycle expansion (see box 2 in Figure S1C).

Alternative entrainment waveform and amplitudes

The entrainment pulse amplitude was defined in such a way that the resulting entrainment region resembles the one observed in rat locomotor activity under light pulse entrainment (24 ± 2 h) [1]. Phase response curves (PRCs) allow the classification of pulse strengths. A PRC describes the magnitude and direction of the phase shifts of an overt rhythm as a function of the time at which the perturbation is presented [2]. Small perturbations lead generically to a smooth PRC (type 1), whereas large pulses lead to discontinuous PRCs of type 0 [3]. In this framework, a pulse amplitude of 0.8 can be classified as a relatively large perturbation, but our results are qualitatively similar with small pulse amplitudes of 0.4 and for sinusoidal perturbations (see Figure S2).

Synchronization of coupled oscillators

Entrainment can be regarded as a particular case of coupled oscillators subject to unidirectional coupling. Therefore, similar features observed in our results might be expected in other synchronization scenarios. As a proof of concept, we studied the time to synchronization of two mutually coupled oscillators. We numerically calculated the time to synchronization of two coupled “sloppy” oscillators as a function of their waveform transition from sinusoidal to spike-like (see Figure S3A). The time to synchronization is significantly reduced as the waveform changes from sinusoidal to spike-like. Further, we calculated the time to synchronization of two coupled sinusoidal oscillators as a function of their radial relaxation transition from a “sloppy” to a “rigid” oscillator (see Figure S3B). In this case, the time to synchronization is reduced as we reduce the radial relaxation time $\tau_r = \frac{1}{\lambda}$. However, a more detailed characterization of the time to synchronization for a system of coupled oscillators is beyond the scope of this paper. In our simulations, the time to synchronization was defined as the time in which both oscillators reach a stable phase difference (less than 5 minutes for a 24 h oscillator). Each point in figure S3 is the mean time to synchronization calculated from 24 different temporal phase initial conditions. The oscillator model is described by Equations 3 and 4 without forcing and with a pulse-like coupling in the y coordinate described by the following equations:

$$\frac{dx_i}{dt} = \frac{T_\mathrm{f}}{24}\left\{\lambda \cdot x_i\left[\frac{1}{\sqrt{x_i^2+y_i^2}}-1\right] - y_i\left[\epsilon\left(\frac{1}{2}+\frac{x_i}{x_i^2+y_i^2}\right)+\mathrm{offset}\right]\right\}, \tag{S3}$$

$$\frac{dy_i}{dt} = \frac{T_\mathrm{f}}{24}\left\{\lambda \cdot y_i\left[\frac{1}{\sqrt{x_i^2+y_i^2}}-1\right]+x_i\left[\epsilon\left(\frac{1}{2}+\frac{x_i}{x_i^2+y_i^2}\right)+\mathrm{offset}\right]\right\}+\frac{k}{1+e^{-\alpha\cdot x_j}} \tag{S4}$$

In order to model a pulse-like saturated coupling, we choose the logistic function with the coupling strength k and the coupling pulse slope α. For Figure S3 we used $k = 0.1$ and $\alpha = 50$.

References

1. Cambras T, Chiesa J, Araujo J, Dez-Noguera A (2004) Effects of photoperiod on rat motor activity rhythm at the lower limit of entrainment. J Biol Rhythms 19: 216–225.

2. Granada A, Hennig RM, Ronacher B, Kramer A, Herzel H (2009) Phase response curves elucidating the dynamics of coupled oscillators. Methods Enzymol 454: 1–27.

3. Winfree A (1980) The geometry of biological time. Springer-Verlag, New York.
