# Supplementary figures and images for: How to Achieve Fast Entrainment? The Timescale to Synchronization
Source: PLoS One. 2009 Sep 23;4(9):e7057. doi: 10.1371/journal.pone.0007057 (PMC2745570; doi:10.1371/journal.pone.0007057)

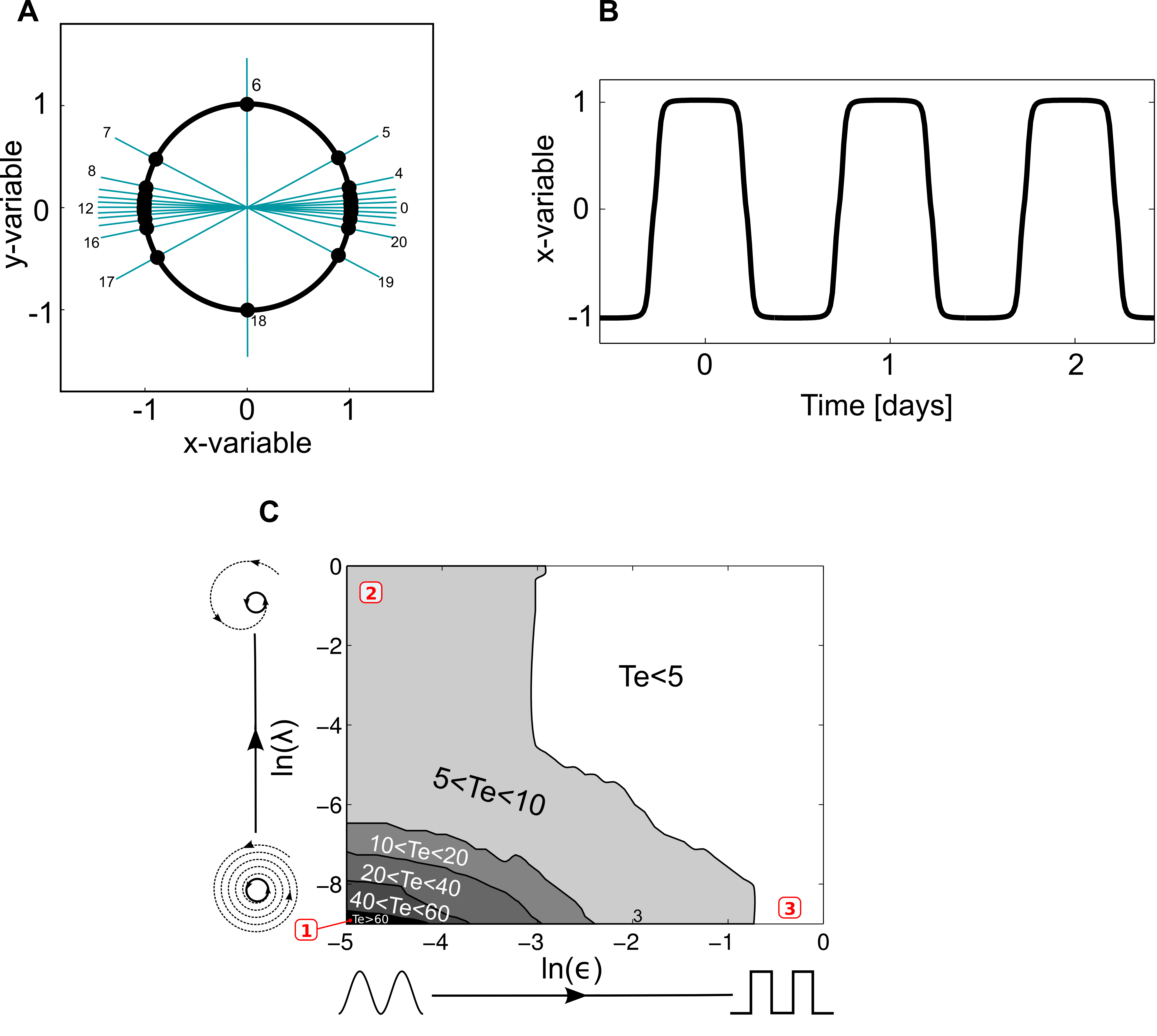

Supplement: Figure S1 — Square waveform oscillator, its time series and the median time to entrainment. (A) Square waveform oscillator: limit cycle with 24 marked phase points (dots) and isochrons (rays). The intersection of each isochron with the limit cycle determines the phase and (B) the temporal evolution of x variable with parameters ε = 1, offset = 0.02, n = 0 and (C) the median time to entrainment as a function of the phase velocity around the limit cycle, ε, and radial relaxation constant, λ, for pulse entrainment. Gray scales refer to the median time to entrainment, where black represents long and white short . (0.72 MB TIF) [file pone.0007057.s002.tif]

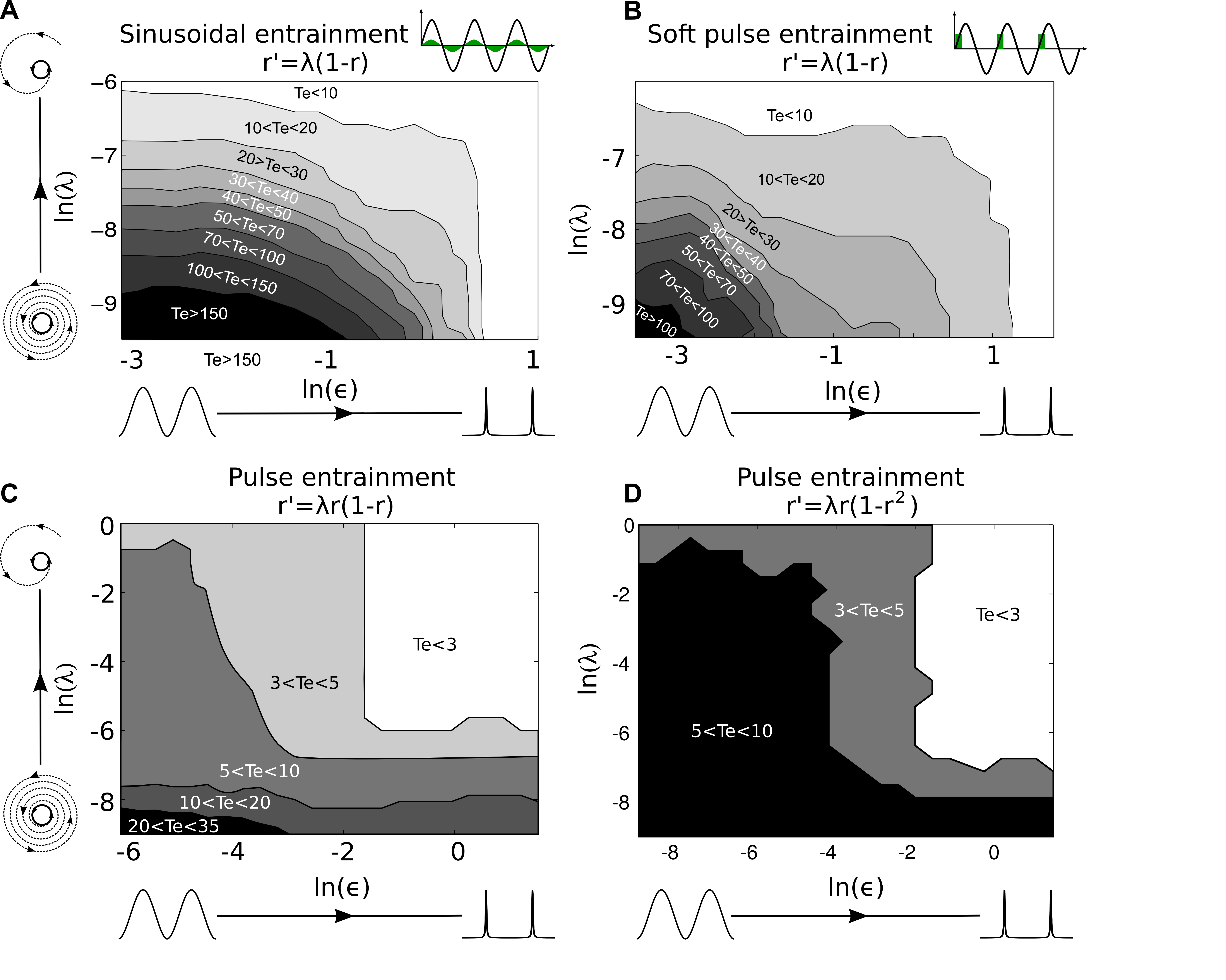

Supplement: Figure S2 — Median time to entrainment for different entrainment signals and oscillators, under soft-pulses entrainment and under medium-sized-pulses for a nonlinear oscillator and for a Hopf oscillator. (A) Entrainment under sinusoidal perturbations with amplitude 0.05. (B) Entrainment under pulse perturbation with amplitude 0.4. (C) Entrainment under 1 h pulse perturbation with amplitude 0.8 for a nonlinear radial relaxation oscillator. (D) Entrainment under 1 h pulse perturbation with amplitude 0.8 for a Hopf oscillator. The median time to entrainment is plotted as a function of the phase velocity around the limit cycle, ε, and radial relaxation constant, λ. Gray scales refer to the median time to entrainment, where black represents long and white short . Both axes are plotted using logarithmic scales.} (1.23 MB TIF) [file pone.0007057.s003.tif]

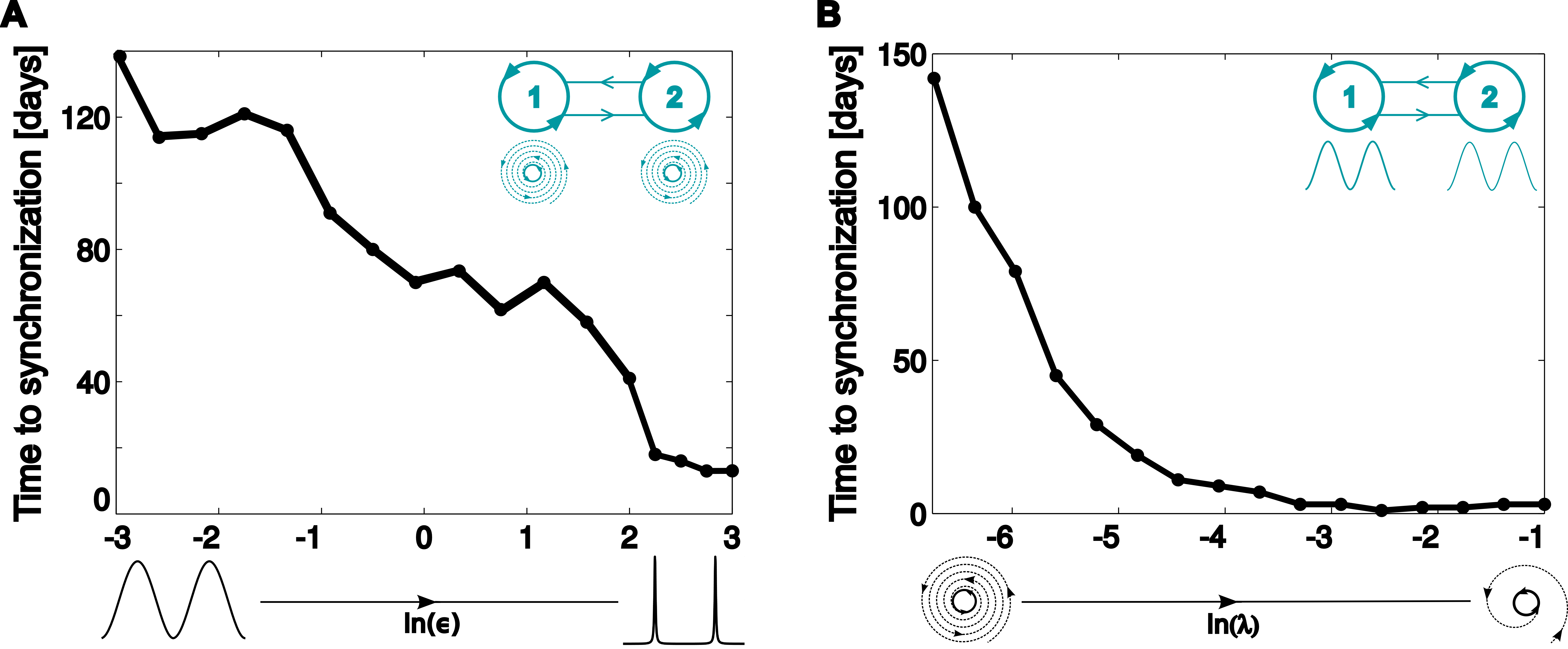

Supplement: Figure S3 — Time to synchronization for two coupled oscillators. (A) Time to synchronization of two coupled “sloppy’ oscillators as a function of their transition from sinusoidal to a spike-like oscillator (B) Time to synchronization of two sinusoidal oscillators as a function of their transition from “sloppy’ to “rigid’ oscillator. See Supporting Information for model details.} (0.83 MB TIF) [file pone.0007057.s004.tif]
